# Impact of type 2 diabetes mellitus on kidney transplant rates and clinical outcomes among waitlisted candidates in a single center European experience

**DOI:** 10.1038/s41598-020-78938-3

**Published:** 2020-12-15

**Authors:** Caterina Dolla, Erika Naso, Alberto Mella, Anna Allesina, Roberta Giraudi, Maria Cristina Torazza, Silvia Bruna Vanzino, Ester Gallo, Antonio Lavacca, Fabrizio Fop, Luigi Biancone

**Affiliations:** 1 Renal Transplantation Center, “A. Vercellone”, Division of Nephrology Dialysis and Transplantation, Department of Medical Sciences, Città della Salute e della Scienza Hospital and University of Turin, Corso Bramante, 88-10126 Turin, Italy; 2 Immunogenetic and Transplant Biology Center, Department of Medical Sciences, Città della Salute e della Scienza Hospital and University of Turin, Turin, Italy

**Keywords:** Nephrology, Kidney diseases, Renal replacement therapy

## Abstract

Despite type 2 diabetes mellitus (T2D) is commonly considered a detrimental factor in dialysis, its clear effect on morbidity and mortality on waitlisted patients for kidney transplant (KT) has never been completely elucidated. We performed a retrospective analysis on 714 patients admitted to wait-list (WL) for their first kidney transplant from 2005 to 2010. Clinical characteristics at registration in WL (age, body mass index -BMI-, duration and modality of dialysis, underlying nephropathy, coronary artery -CAD- and/or peripheral vascular disease), mortality rates, and effective time on WL were investigated and compared according to T2D status (presence/absence). Data about therapy and management of T2D were also considered. At the time of WL registration T2D patients (n = 86) were older than non-T2D (n = 628) (58.7 ± 8.6 years vs 51.3 ± 12.9) with higher BMI (26.2 ± 3.8 kg/m^2^ vs 23.8 ± 3.6), more frequent history of CAD (33.3% vs 9.8%) and peripheral vascular disease (25.3% vs 5.8%) (*p* < 0.001 for all analyses). Considering overall population, T2D patients had reduced survival vs non-T2D (*p* < 0.001). Transplanted patients showed better survival in both T2D and non-T2D groups despite transplant rate are lower in T2D (75.6% vs 85.8%, *p* < 0.001). T2D was also associated to similar waiting time but longer periods between dialysis start and registration in WL (1.6 years vs 1.2, *p* = 0.008), comorbidity-related suspension from WL (571 days vs 257, *p* = 0.002), and increased mortality rate (33.7% vs 13.9% in the overall population, *p* < 0.001). In T2D patients admitted to WL, an history of vascular disease was significantly associated to low patient survival (*p* = 0.019). In conclusion, T2D significantly affects survival also on waitlisted patients. Allocation policies in T2D patients may be adjusted according to increased risk of mortality and WL suspension due to comorbidities.

## Introduction

Type 2 diabetes mellitus (T2D) represents a real public health problem according to its increasing incidence and the severity of the disease with multi-organic involvement^1^. T2D long-term complications include disability, reduced quality of life, premature death, and end-stage kidney disease (ESKD)^2^ with a consequent spreading percentage of dialytic patients affected by T2D^3^. In this population a multi-specialistic approach to prevent and manage T2D-related comorbidity is mandatory. Despite improvements over the last decade have leaded to an amelioration in survival rates, both diabetes and ESKD increased risk of mortality, particularly due to cardiovascular complications, especially in cases who needed renal-replacement therapies^2,4^.

KT is considered the gold standard for ESKD for its superiority to dialysis for all outcomes (quality of life, overall survival, economic costs)^5,6^ and, despite some authors suggested negative or “not-so-positive” results in T2D patients^7^, recent reports outline favorable outcome also in T2D patients^8^. On the other hand few studies are focused on T2D patients who could be considered eligible for KT, and none of them at the best of our knowledge have evaluated if WL time—a variable directly correlated to negative outcome both for patient and KT^7,8^—could be influenced by the intrinsic T2D frailty for T2D-related comorbidities.

In this study we investigate the effect of T2D on morbidity and mortality on waitlisted patients also comparing the effective time on WL according to T2D status (presence/absence).

## Results

### Baseline characteristics

During the investigated period, 714 patients were registered in our WL and 86 out of 714 (12%) were affected by T2D. Clinical characteristics are summarized in Table 1.

**Table 1:** Characteristics and follow-up of studied population according to T2D presence/absence.

	T2D (n = 86)	Non-T2D (n = 628)	Total	*p*
Age, years	58.7 ± 8.6	51.3 ± 12.9	52.2 ± 12.2	< 0.001
Sex (M/F ratio)	61/25	389/239	450/264	0.065
BMI, kg/m^2^	26.2 ± 3.8	23.8 ± 3.6	24 ± 3.6	< 0.001
Coronary artery disease, n (%)	25 (33.3)	56 (9.8)	81 (12.5)	< 0.001
Periferic vascular disease, n (%)	19 (25.3)	36 (6)	55 (7.4)	< 0.001
Kidney Transplantation, n (%)	65 (75.6)*	539 (85.8)*	604 (84.6)*	0.013
Living donors, n (%)	2/65 (3.1)	24/539 (4.4)	26/604 (4.3)	0.06
Donor age, years	62.2 ± 14.2	56.9 ± 15	58.1 ± 14.5	0.006
Death				
Overall population, n (%)	29 (33.7)	87 (13.9)	116 (16.2)	< 0.001
No KT, n (%)	15 (71.4)*	43 (48.3)*	58 (52.7)*	0.047
Pre-transplant dialysis time, years	3.8 ± 2.5	4.7 ± 3.6	4.6 ± 3.7	0.18
Period of WL suspension, days	571 (289–1073)	257 (83.5–802)	286 (92.7–860.7)	0.002
Time between dialysis start and WL registration, years	1.6 (1.1–2.5)	1.2 (0.72–2.2)	1.3 (0.75–2.3)	0.008

Data are expressed as mean ± SD or median (25°–75° percentile) according to their distribution.

*Calculated on the total for each single group.

Briefly, at the admission patients in T2D group were older than non-T2D (58.7 ± 8.6 years vs 51.3 ± 12.9) with higher BMI (26.2 ± 3.8 kg/m^2^ vs 23.8 ± 3.6), more frequent history of CAD (33.3% vs 9.8%) and peripheral vascular disease (25.3% vs 5.8%) (*p* < 0.001 for all analyses). No differences were observed regarding dialysis length and modality.

Pharmacological treatment of T2D and cardiovascular characteristics of our T2D patients admitted to WL were reported in Table 2.

**Table 2:** Pharmacological and cardiovascular characteristics of T2D group.

	T2D (n = 86)
Insulin therapy, n (%)	48 (55.8%)
Oral anti-diabetic drugs, n (%)	5 (5.8%)
Diet alone, n (%)	22 (25.6%)
Not specified, n (%)	11 (12.8%)
Left ventricular hypertrophy, n (%)	50 (58.1%)
PCI, n (%)	13 (15.1%)
CABG, n (%)	4 (4.7%)
Cerebrovascular accident, n (%)	9 (10.5%)
HbA1c, mmol/mol *	50 (40–61)
Ejection fraction, % *	60 (55–64)

*Data are expressed as mean ± SD or median (25°–75° percentile) according to their distribution.

*PCI* percutaneous coronary intervention, *CABG* coronary artery bypass grafting, *HbA1c* glycosylated haemoglobin.

In detail, 48 out of 86 patients (55.8%) received insulin therapy, 5/86 (5.8%) were treated with oral anti-diabetic drugs, and 22/86 (25.6%) followed diet alone. HbA1c value at the time of registration in WL were within the correct range of glycemic control [median HbA1c 50 mmol/mol (25° 40–75° 61)].

Despite a more frequent history of CAD and evidence of left ventricular hypertrophy in the majority of T2D patients (50/86, 58.1%), echocardiography assessment revealed a normal median ejection fraction [median 60% (25° 55–75° 64)]. Considering vascular disease, percutaneous coronary intervention (PCI) and coronary artery bypass grafting (CABG) were performed in 13/86 (15.1%) and 4/86 (4.7%) respectively; an history of cerebrovascular accident was also observed in 9/86 T2D patients (10.5%).

During the follow-up (mean 8.4 ± 3 years) 604 patients underwent KT (84.6%) with a significantly lower KT rate in T2D group (75.6% vs 85.8%, *p* = 0.013). The number of living donors was similar in each group but, in deceased donors, donor’s age was higher in T2D group.

### Survival analysis

**Figure 1:**
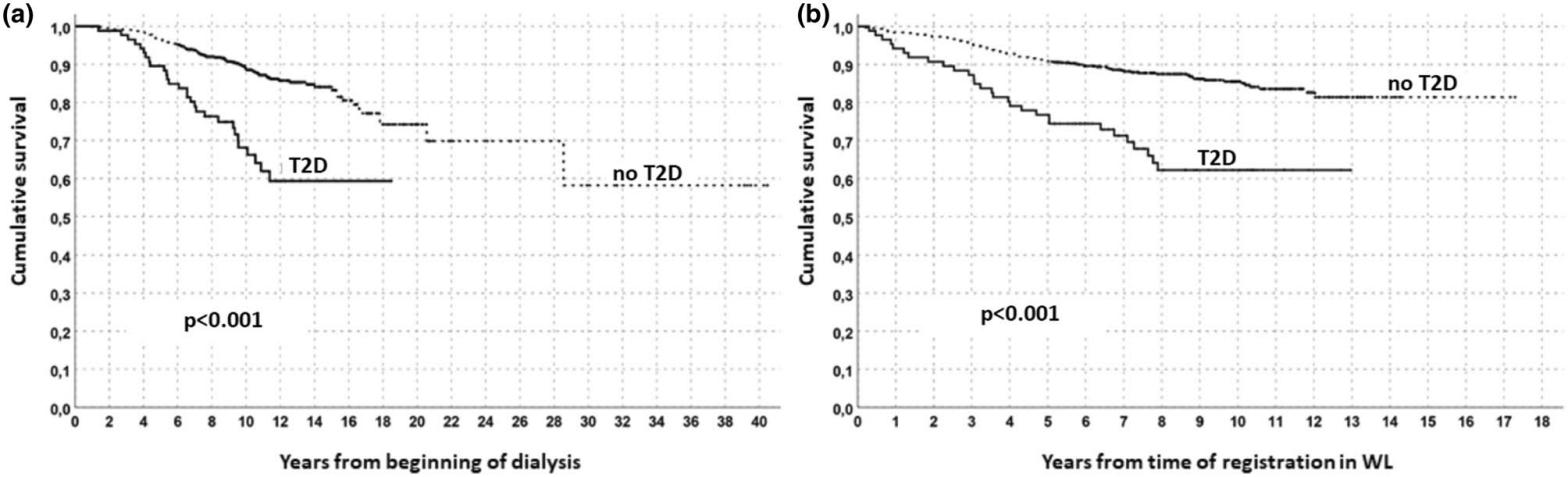
Survival analysis in the overall population (Kaplan–Meyer) according to T2D presence/absence. Follow/up start in (**a**) at the beginning of dialysis or in (**b**) at time of registration in WL.

**Figure 2:**
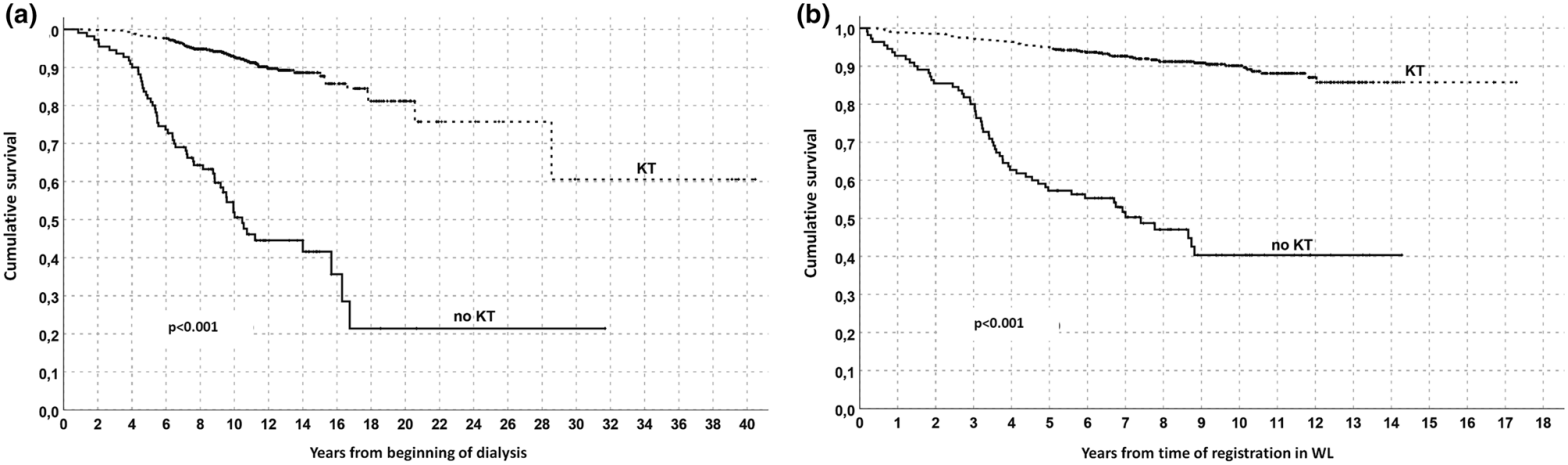
Survival analysis in the overall population (Kaplan–Meyer) according to kidney transplantation or maintenance on WL. Follow/up start in (**a**) at the beginning of dialysis or in (**b**) at time of registration in WL.

**Figure 3:**
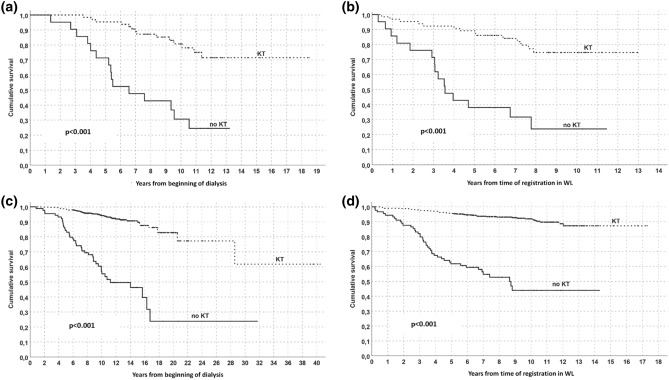
Survival analysis in T2D (**a**, **b**) and non-T2D (**c**, **d**) group (Kaplan–Meyer) according to kidney transplantation or maintenance on WL. Follow/up start in (**a**) and (**c**) at the beginning of dialysis or in (**b**) and (**d**) at time of registration in WL.

Survival rates were investigated both considering the f/up starting at the beginning of dialysis or at the time of registration in WL. Patients in T2D group had a reduced survival than non-T2D (Fig. 1 a–b) and transplanted patients had a better survival than those who remain in WL (Fig. 2 a–b); comprehensively, all transplanted patients had a better survival than those remaining on WL. This favorable outcome of KT patients was confirmed in the intra-group analysis both for T2D and non-T2D groups, despite T2D showed lower survival rates (Fig. 3).

Analyzing the possible impact of dialysis duration on survival we noted that patients who died during the f/up have a longer dialysis history before the registration in WL [19.8 months (25° 10–75° 34.5) vs 15 months (25° 8.9–75° 26.2), *p* < 0.01]; stratifying this data to patients who underwent KT this variable remained significant in the overall population and in non-T2D group.

Among both the overall population and the subgroup of patients who did not receive KT the mortality rate was significantly higher in T2D (Table 1); focusing on this group (which is not biased by transplant-related events) cardiovascular events and infections, as expected, were the most common causes of death in both T2D and non-T2D (Table 3). Cox regression analysis showed a 2.4 fold higher mortality risk (OR 2.4, IC 95% 1.5–3.6) in T2D vs non-T2D group, 7.5 fold higher (OR 7.5, IC 95% 5.2–10.7) in patients who remained in WL vs KT, and 18.5 fold higher (OR 18.5, IC 95% 4.9–10.1) for the group with lower survival (non-transplanted patients in T2D group) vs patients with favorable one (non-T2D who underwent KT).

**Table 3:** Cause of death in waitlisted patients who not received KT according to T2D presence/absence.

	T2D (n = 15)	Non-T2D (n = 43)	*p*
Cardiovascular events, n (%)	8 (53.3)	19 (44.2)	0.455
Infection, n (%)	4 (26.7)	11 (25.6)	
Tumors, n (%)	2 (13.3)	2 (4.7)	
Other, n (%)	1 (6.7)	6 (14)	
Not specified, n (%)	0 (0)	5 (11.6)	

A stratification of survival analysis in T2D group revealed the significant negative impact of an history of vascular accident (intended as PCI, CABG and/or cerebrovascular accident) on this population (Supplementary Figure S1, *p* = 0.015); also insulin therapy and a worst glycemic control showed a negative trend on patients survival (Supplementary Figures S2 and S3).

### Waiting list period

The median waiting time for KT was not statistically different between T2D and non-T2D group [466.5 days (25° 104.5–75° 1182) vs 620 (25° 174–75° 1619) respectively, *p* = 0.095].

Despite the percentage of patients who experienced at least one temporary suspension from the WL was quite similar in T2D and non-T2D (36% vs 39.7% respectively; *p* = 0.3), the period of suspension was longer in T2D group (571 days, 25° 289–75° 1073 vs 257, 25° 83.5–75° 802; *p* < 0.001). T2D group also experienced an higher percentage of patients definitively dropped out from WL for clinical reasons (17.4% vs 8.4%, *p* = 0.008) and a longer time elapsed between dialysis start and WL registration (1.6 years, 25° 1.1–75° 2.5 vs 1.2, 25° 0.72–75° 2.2; *p* = 0.008) (Table 1).

## Discussion

T2D is now considered the leading cause of ESKD in western countries, with a percentage of affected patients that range from 30% in European area^2,9^ to 45% in USA^10,11^. In our population the prevalence of T2D in dialytic population significantly increased over time, from 25% (2004–2008) to 29.1% (2009–2013)^2^. In subjects who ultimately needed renal-replacement therapies, T2D also negatively affect patient survival^12^. In 2009 Report of United States Renal Data system the 10-year survival of T2D patient on hemodialysis stands around 10%^13^; in another study in France 32% of patients with T2D died after a mean f/up of about 7 months^14^.

Despite its increasing incidence, the proportion of T2D patients admitted to WL is relatively low (ranging from 15–20% in Europe to about 30% in USA) as reported in literature^5,15–20^, and confirmed in our experience.

The prevalence of T2D among dialytic population in our region where KT Center is located (Piemonte e Valle d’Aosta) is about 29%^2^; however, considering the average age of patients who started dialysis (≈ 70 years)^21^, it is conceivable that only a portion of these T2D patients would be considered eligible for KT.

This difference is probably referred to T2D-related comorbidities (i.e. CAD, peripheral vascular disease, infections) who may determine an a-priori exclusion or a delay/interruption in WL process for acute clinical events (i.e. conditions requiring surgical or angiographic procedures as coronary artery intervention). A demonstration derived from the paper by Villar et al.^5^ where T2D and elderly age (> 60 years) are identified as the leading causes for WL exclusion, and both elderly and diabetic patients need a longer time for the pre-transplantation balance.

Data are still lacking about morbidity and mortality in the subgroup of T2D patients considered eligible for WL. Schold et al.^22^ reported that T2D patients are more prone to be removed from WL and Ningyan et al.^23^ underlined that a great proportion of patients were dropped-out from WL due to a cardiovascular event, a condition extensively related to T2D.

In our analysis, as expected, T2D patients at the time of WL registration were older, with higher BMI and more frequent history of CAD and peripheral vascular disease. Despite the pre-admission balance demonstrates that the majority of patients had a normal ejection fraction and were on-target for HbA1c according to international guidelines^24^, our adjunctive analysis revealed the significant role of an history of vascular accident on reducing patient survival, with also a univocal trend for patients with poor glycemic control: all these information stressed the importance of good T2D management also in the subgroup of patients admitted to WL.

As also expected, KT determined a significant outcome amelioration in both T2D and non- T2D group. Many studies showed similar results^4,25^ also suggesting an adjunctive benefit for pre-emptive transplantation^26,27^. Despite slightly better results were observed in non-T2D group, as outlined by Cosio et al.^6^ mortality in KT recipients with T2D has progressively declined over time, thanks to the improvements in the management of both T2D and KT, and the beneficial effect of KT as also observed in our population is overwhelming. In accordance with K-DOQI guidelines all ESKD patients, especially those with T2D, need to be referred to a nephrological center also for a prompt pre-transplant evaluation^28^.

On the other hand, despite patients in T2D and non-T2D group have a similar median WL-time and percentage of temporary drop-out from WL, transplant rate was lower in T2D patients; moreover, T2D experienced a threefold higher mortality after waitlisting which remained extremely high in not transplanted patients, suspension periods were longer and associated with a higher definitive drop-out rate in T2D group suggesting a significant negative impact of T2D-related comorbidities. Previous studies identify T2D and cardiovascular disease as major dominants of lower odds of being on the WL^29–31^.

Similar to Lee et al.^8^ T2D patients experienced a higher mortality during WL also in our study and, as expected, cardiovascular events (probably underestimated due to the absence of a determined death cause in the majority of patients) were the most common cause of death in this group. Cardiovascular disease are typical comorbidities affecting all ESKD patients, with higher prevalence and incidence than in general population: they can aggravate the underlying medical condition and limit the access to KT^7^. Successful KT accords major benefits by reducing cardiovascular risk in these patients, and efforts are needed to minimize WL time^32^.

Based on our study design, not influenced by a negative-selection bias, we speculate that the reduced likelihood of KT among T2D group may also suggest a per-se negative effect of T2D on KT rate. In one recent study by Jeon et al. the occurrence of cardiovascular disease but not diabetes determined lower transplant rates^7^, so it could be important to investigate the possible negative T2D effect in larger cohorts.

Despite the obvious limitations (low percentage of T2D patients admitted to WL, retrospective design) which also affected the majority of the reported experiences in this area, our study demonstrates that (a) T2D significantly affects patients survival also on patients who are eligible for WL (b) T2D patients, probably due to T2D-related conditions (especially vascular disease), have a reduced probability of being transplanted (c) KT also in T2D patients is clearly associated with better survival. In our opinion efforts are needed in order to estimate as earlier as possible the eligibility of T2D patients for WL and, at the same time, allocation policies may be adjusted according to the increased risk of mortality and WL suspension due to comorbidities in T2D patients.

## Methods

Retrospective study on the waitlisted population of our center (Renal Transplantation Center “A. Vercellone”, Turin, Italy) including all patients who are considered eligible and included in active WL in the period between January 2005 and December 2010, and transplanted until February 2016. The analysis included patients from two regions of northern Italy (Piemonte and Valle d’Aosta) and at first KT; type 1 and post-transplant diabetes mellitus, and patients with glycated hemoglobin (HbA1c) > 64 mmol/mol at the time of inclusion in WL were excluded. Diagnosis of T2D was made according to the American Diabetes Association recommendations^24^. Recorded variables at registration in WL included age, sex, dialysis duration and modality (hemodialysis or peritoneal dialysis), body mass index (BMI), history of coronary artery (CAD) and/or peripheral vascular disease. All these conditions were compared to T2D presence/absence. Data were collected from the database of our transplantation center (ITR02, Dialysis and Transplantation Registry of Piemonte) and are thereafter elaborated in anonymous format. This study is covered by Ethical Committee approval, resolution number 1449/2019 on 11/08/2019 ("TGT" observational study).

Statistical analysis was performed with SPSS (IBM SPSS Statistics, vers.25.0.0). Continuous variables are presented as mean ± standard deviation or as median (25°-75° percentile), according to their distribution analysed with Kolmogorov–Smirnov test. The difference between groups was analysed, respectively, with t-test or Mann–Whitney test. Some cut-off levels were with ROC curves. Categorical variables are presented as fraction and Pearson’s or, for small samples, Fisher’s exact test was employed to compare groups. The odds ratios (OR) with 95% Confidence Interval were used as a measure of relative risk. Univariate Survival analysis was performed by means of the Kaplan–Meier method with Log Rank test to compare strata. Cox proportional-hazards model was used to investigate the association between the survival time of patients and predictor variables. Significance level for all tests was set at *p* < 0.05.

### Ethical statement

This study is covered by Ethical Committee approval, Resolution Number 1449/2019 on 11/08/2019 ("TGT" observational study) for which all patients sign an informed consent. The study was conducted in accordance with Declaration of Helsinki.

## Supplementary information

Supplementary Information 1.

## Data Availability

All data and datasets used and/or analysed during the current study are available from the corresponding author on reasonable request.
