# Supplementary material for: Impact of type 2 diabetes mellitus on kidney transplant rates and clinical outcomes among waitlisted candidates in a single center European experience
Source: Sci Rep. 2020 Dec 15;10:22000. doi: 10.1038/s41598-020-78938-3 (PMC7738492; doi:10.1038/s41598-020-78938-3)

**Supplementary information to:**

**Impact of type 2 diabetes mellitus on kidney transplant rates and clinical outcomes among waitlisted candidates in a single center European experience**

**Authors**

Caterina Dolla^1^ MD, PhD, Erika Naso^1^ MD, Alberto Mella^1^, MD, PhD, Anna Allesina^1^ MD, Roberta Giraudi^1^ MD, Maria Cristina Torazza^1^ MD, Silvia Bruna Vanzino^2^ MD, Ester Gallo^1^ MD, Antonio Lavacca^1^ MD, Fabrizio Fop^1^ statistic, Luigi Biancone^1*^ Prof., MD, PhD

^1^ Renal Transplantation Center, “A. Vercellone”, Division of Nephrology Dialysis and Transplantation, Città della Salute e della Scienza Hospital and Department of Medical Sciences, University of Turin, Italy

^2^ Immunogenetic and Transplant Biology Center, Città della Salute e della Scienza Hospital and Department of Medical Sciences, University of Turin, Italy

Content:

Page 2: Survival analysis in T2D group (Kaplan-Meyer) according to vascular disease presence/absence.

Page 3: Survival analysis in T2D group (Kaplan-Meyer) according to Hb1Ac.

Page 4: Survival analysis in T2D group (Kaplan-Meyer) according to T2D therapy.

**Figure S1**. **Survival analysis in T2D group (Kaplan-Meyer) according to vascular disease presence/absence.** Vascular disease is intended as history of PCI, CABG and/or cerebrovascular accident. Follow/up start at time of registration in WL.

**
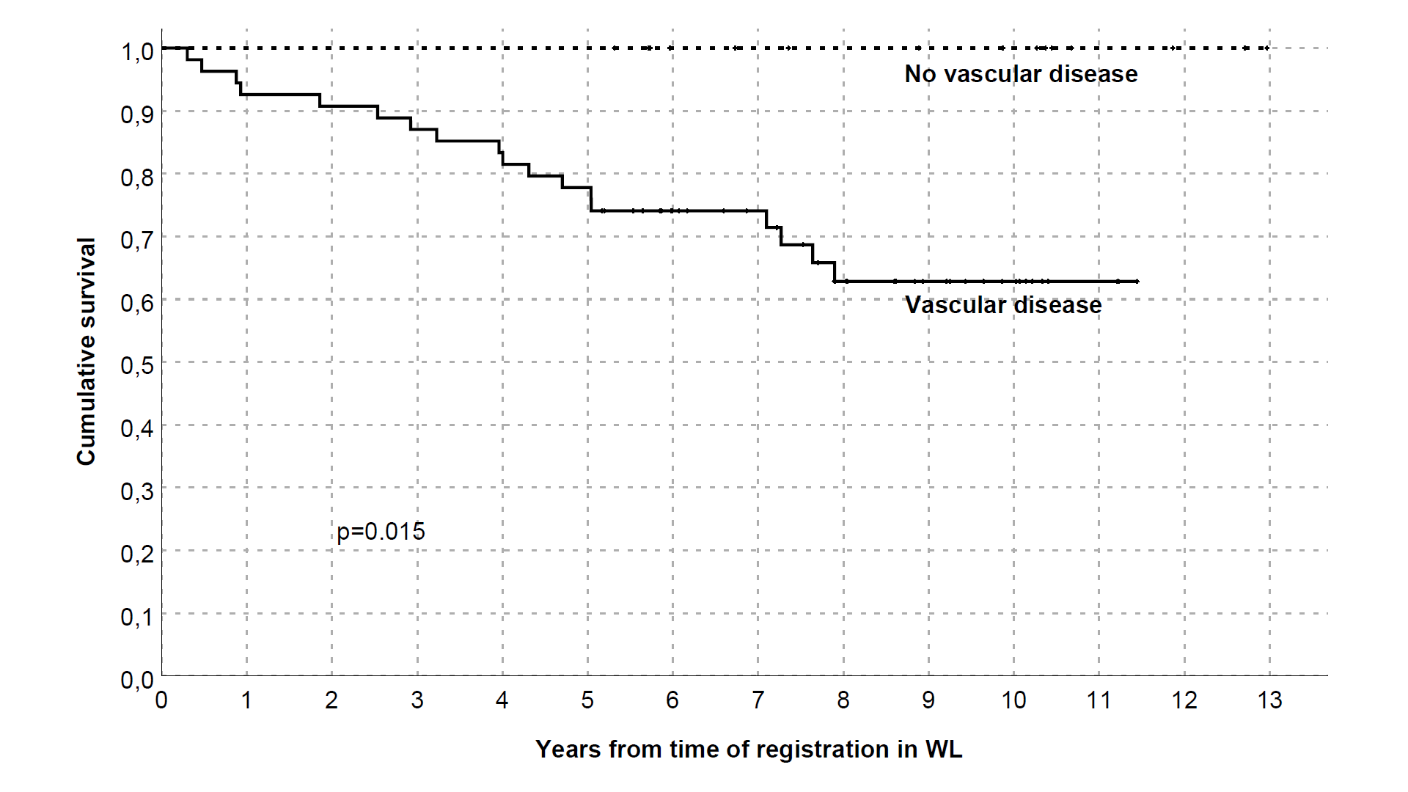
**

**Figure S2**. **Survival analysis in T2D group (Kaplan-Meyer) according to Hb1Ac.** Follow/up start at time of registration in WL.

**
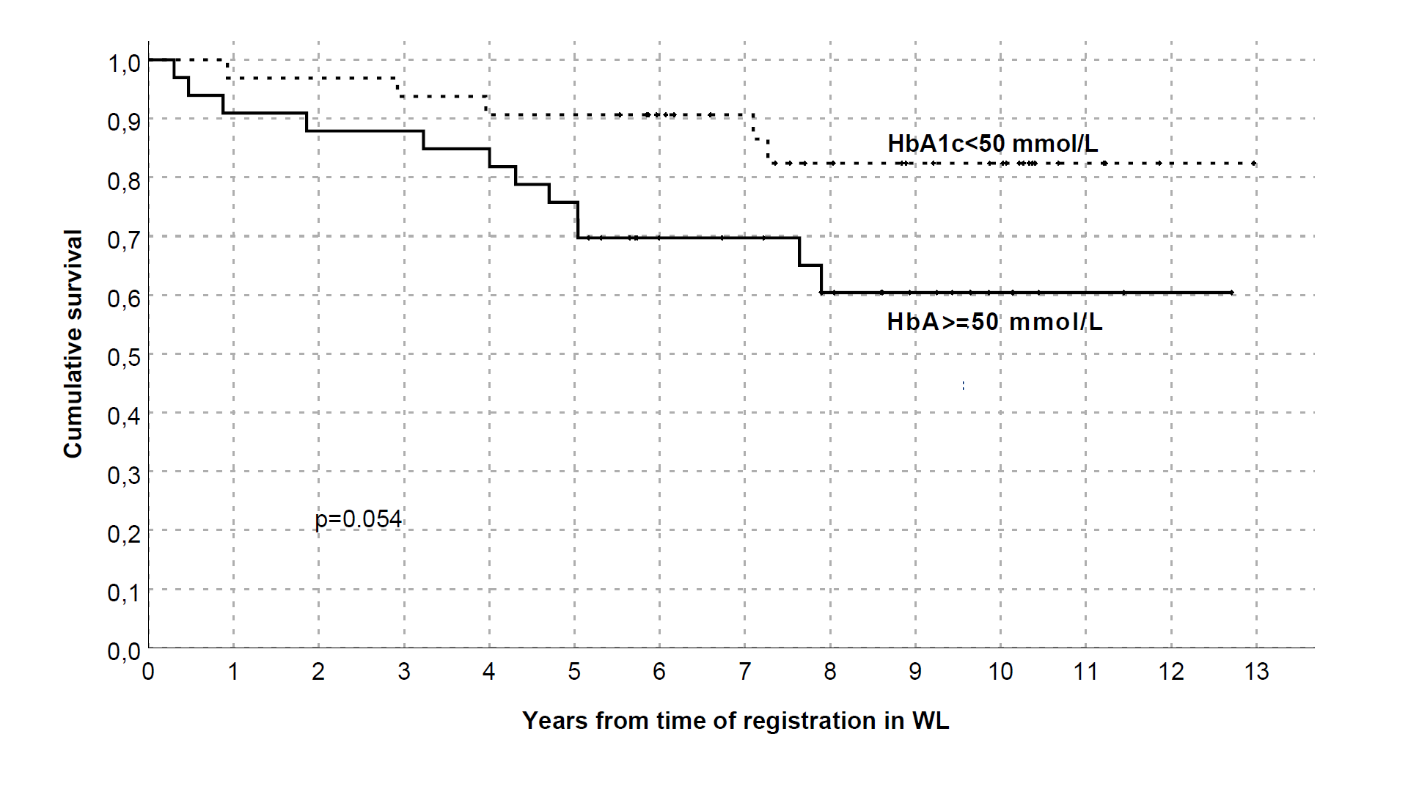
**

**Figure S3**. **Survival analysis in T2D group (Kaplan-Meyer) according to T2D therapy.** Follow/up start at time of registration in WL.


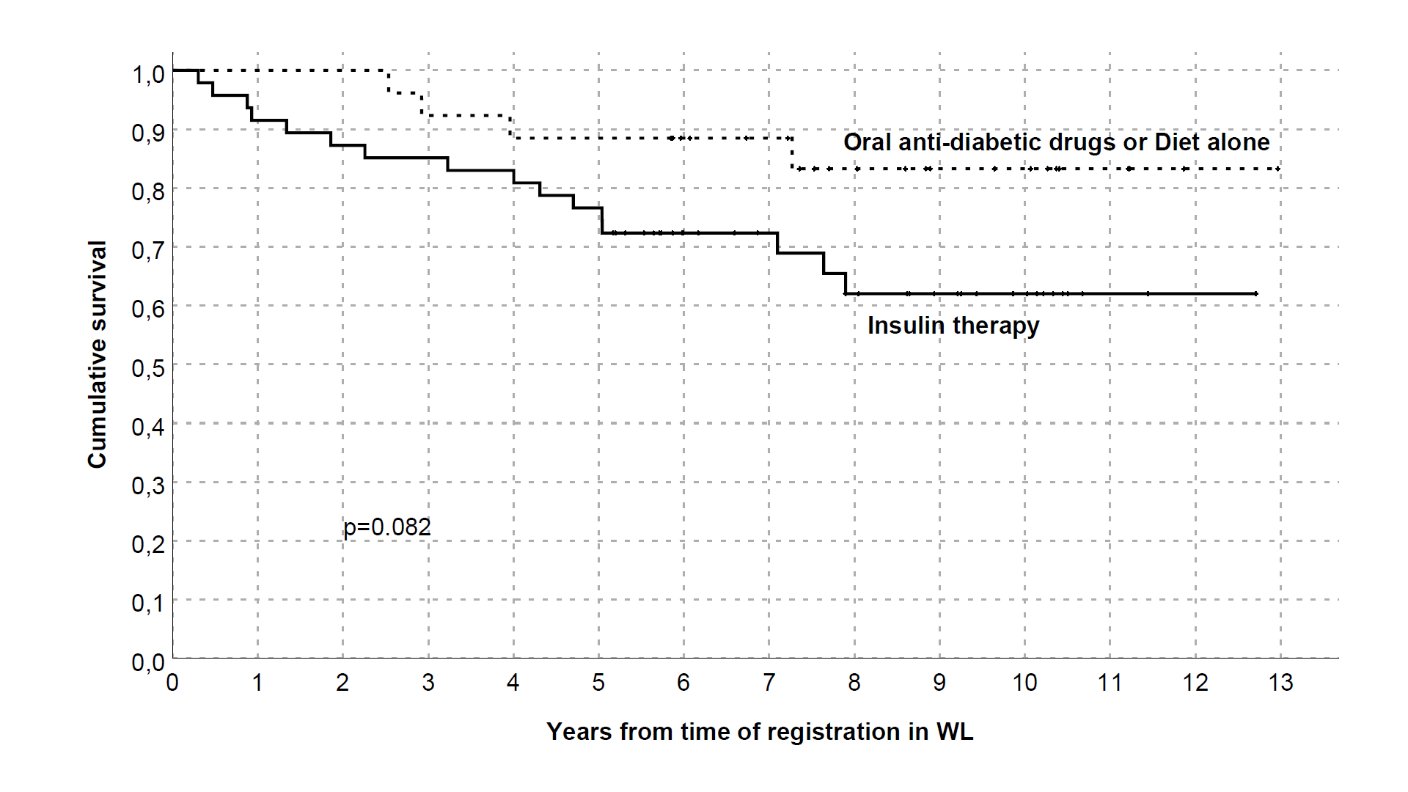

Supplement: Supplementary file 1 — Supplementary Information 1. [file 41598_2020_78938_MOESM1_ESM.docx]
